# Accurate LAI estimation of soybean plants in the field using deep learning and clustering algorithms

**DOI:** 10.3389/fpls.2024.1501612

**Published:** 2025-01-22

**Authors:** Bing Shi, Luqi Guo, Lejun Yu

**Affiliations:** National Key Laboratory for Tropical Crop Breeding, Sanya Research Institute of Hainan University, Hainan University, Sanya, China

**Keywords:** UAV, LiDAR, high-throughput, soybean, machine learning, PointNet++

## Abstract

The leaf area index (LAI) is a critical parameter for characterizing plant foliage abundance, canopy structure changes, and vegetation productivity in ecosystems. Traditional phenological measurements are often destructive, time-consuming, and labor-intensive. This paper proposes a high-throughput 3D point cloud data processing pipeline to segment field soybean plants and estimate their LAI. The 3D point cloud data is obtained from a UAV equipped with a LiDAR camera. First, The PointNet++ model was applied to simplify the segmentation process by isolating field soybean plants from their surroundings and eliminating environmental complexities. Subsequently, individual segmentation was achieved using the Watershed approach and k-means clustering algorithms, segmenting the field soybeans into individual plants. Finally, the LAI of soybean plant was estimated using a machine learning method and validated against measured values. The PointNet++ model improved segmentation accuracy by 6.73%, and the watershed algorithm achieved F1 scores of 0.89–0.90, outperforming k-means in complex adhesion cases. For LAI estimation, the SVM model showed the highest accuracy (R² = 0.79, RMSE = 0.47), with RF and XGBoost also performing well (R² > 0.69, RMSE< 0.65). This indicates that the individual segmentation algorithm, Watershed-based approach combined with PointNet++, can serve as a crucial foundation for extracting high-throughput plant phenotypic data. The experimental results confirm that the proposed method can rapidly calculate the morphological parameters of each soybean plant, making it suitable for high-throughput soybean phenotyping.

## 1 Introduction

Soybean (Glycine max L. Merr.) is one of the most important protein and oil crops (Kim et al., 2012). The protein component is the most prominent in soybean seeds (Hartman et al., 2011), while the oil component accounts for about 18-20% of the seed’s dry weight and is widely used for human consumption and various industrial applications (Clemente and Cahoon, 2009). The leaf area index (LAI) is a key determinant of soybean yield, with optimal yields achieved at an LAI value of 3.5-4.0 (Malone et al., 2002). LAI reflects the total leaf area of plants per unit surface area. Traditional destructive sampling to assess LAI is labor-intensive, time-consuming, and often lacks scalability, making it impractical for large-scale or high-throughput measurements. In contrast, our proposed method offers a non-destructive, efficient, and automated approach, significantly reducing labor and time requirements while maintaining high accuracy in large field environments. The capability to accurately and rapidly acquire leaf area index (LAI) is essential for process-based ecological research (Zheng and Moskal, 2009).

The rapid development of UAV technology has enabled the use of UAV-based multispectral imagery to estimate LAI through statistical methods (Hunt et al., 2008). UAVs have proven to be effective remote sensing platforms for monitoring crop conditions on individual farm fields, and UAV-based photogrammetry can generate LiDAR-like 3D point cloud data containing crop structure information (Song et al., 2020). However, studies on plant data analysis have mainly concentrated on separating plant populations into separate individuals. Achieving accurate and high-throughput segmentation of plants in complex datasets, such as distinguishing soil surface features from seedlings, remains a challenging task (Yang et al., 2020). To address these challenges, researchers have developed various approaches, including the use of PointNet deep learning models to segment organs of sorghum plants from radar 3D point cloud data. The segmentation results were used to extract sorghum plant phenotypic traits (Patel et al., 2023). Xie et al.’s hierarchical modeling demonstrated superior performance in point cloud segmentation (Xie et al., 2024). Li et al. showcased the potential of deep learning in phenotypic parameter extraction by applying PointNet for semantic segmentation of maize organs (Li et al., 2022). However, in practical applications, these methods have demonstrated limited efficiency (Saeed and Li, 2021). In the absence of overlap between plants in a population, individual plant segmentation can be achieved through straightforward division methods. However, this approach is not applicable to real-world crop planting and growth conditions, where plant hybridization or adhesion is common. Achieving accurate single plant segmentation in cases where plant leaves overlap remains a significant research challenge. It is a research difficulty to realise individual segmentation in the case of crossed plant leaves. For example, the segmentation of overlapping leaves and individual leaf adhesion cannot be segmented with deep learning methods, and they need to be segmented with clustering segmentation, and they are segmented by a region growing algorithm based on the Multiscale Tensor Voting Method (MSTVM), which is able to produce independent leaves and overlapping leaves (Li et al., 2022; Liu et al., 2022). To address the challenges of individual maize plant segmentation caused by leaf overlap, a combination of Euclidean and K-means clustering based on Euclidean distance was employed. This approach significantly enhanced segmentation outcomes compared to using Euclidean clustering alone (Miao et al., 2023). Hu et al. proposed a point cloud segmentation method combining an improved point transformer and hierarchical clustering, achieving better individual tree segmentation with a MIOU of 0.742 (Hu et al., 2023). Miao et al. successfully achieved individual segmentation by K-means clustering method using point cloud data collected from banana plants, but did not analyse it with respect to segmentation accuracy (Miao et al., 2022). Hui et al. proposed an adaptive kernel bandwidth mean shift segmentation and hierarchical technique for UAV LiDAR individual tree extraction, achieving higher accuracy and completeness than traditional methods, though performance declines in densely clustered trees (Hui et al., 2021). Li et al. developed an automated method for pear tree branch and leaf segmentation using LiDAR point clouds, combining PointNet++ for semantic segmentation and mean shift clustering for individual leaf extraction (Li et al., 2023). Jin used deep learning and region-growing algorithms to separate maize plants, achieving an accuracy of 94% (Jin ShiChao et al., 2018). However, stem and leaf segmentation methods for monocots are well established but do not address how to segment monocots of populations. There is no further research on monocot segmentation methods for large adhering plants in airborne LiDAR crops, so the segmentation effect of plants needs to be analyzed at the point cloud level.

Remote sensing is the only feasible method to invert the leaf area index (LAI) on a large scale or even on a global scale (White et al., 2000). The use of remote sensing data for LAI estimation promises accurate measurements on a large scale. Yang et al. proposed an improved geometry-based method for fisheye-based forest LAI field measurements, incorporating tree height, crown depth, and pixel size, which significantly improved accuracy, reducing RMSE by almost 70% compared to previous methods (Yang et al., 2023). Passive optical remote sensing, which does not require the active emission of signals but instead relies on the reflection or scattering of natural light, offers lower costs and simpler operational methods. It has been widely used in the estimation of Leaf Area Index (LAI) (White et al., 2000; Broge and Leblanc, 2001; Gitelson, 2004). LiDAR is an active remote sensing technology that scans and analyzes the vertical characteristics of surface objects or vegetation, improving the accuracy of ecological parameter estimation such as LAI (White et al., 2000).LiDAR has been applied for inversion of LAI in forests (Broge and Leblanc, 2001; Gitelson, 2004; Viña et al., 2011). The LAI is indirectly estimated by measuring optical characteristics such as the light transmittance and reflectance of the plant canopy (Gitelson, 2004; Tang et al., 2012; Zheng et al., 2012). LAI was estimated for individual sweet corn plants in field experiments using a UAV-based method, with vegetation indices (NDVI, EVI2, and SR) validated, where SR showed the strongest correlation with both yield and LAI estimation (Jung et al., 2023). LiDAR remote sensing captures the 3D structure and physical features of forests, effectively reflecting canopy vertical distribution and foliage density, thereby providing accurate data for LAI estimation (Riaño et al., 2004; Zhao and Popescu, 2009; Zheng et al., 2012; Luo et al., 2015).

In this paper, taking soybean plants with different genes as the research object, We used airborne LiDAR to collect point cloud data of soybean plants and researched individual segmentation methods based on watershed and K-means clustering. Main tasks include: 1) An individual segmentation pipeline is proposed; 2) Precision estimation of Leaf Area Index (LAI) using machine learning.

## 2 Materials and methods

### 2.1 Overview

**Figure 1:**
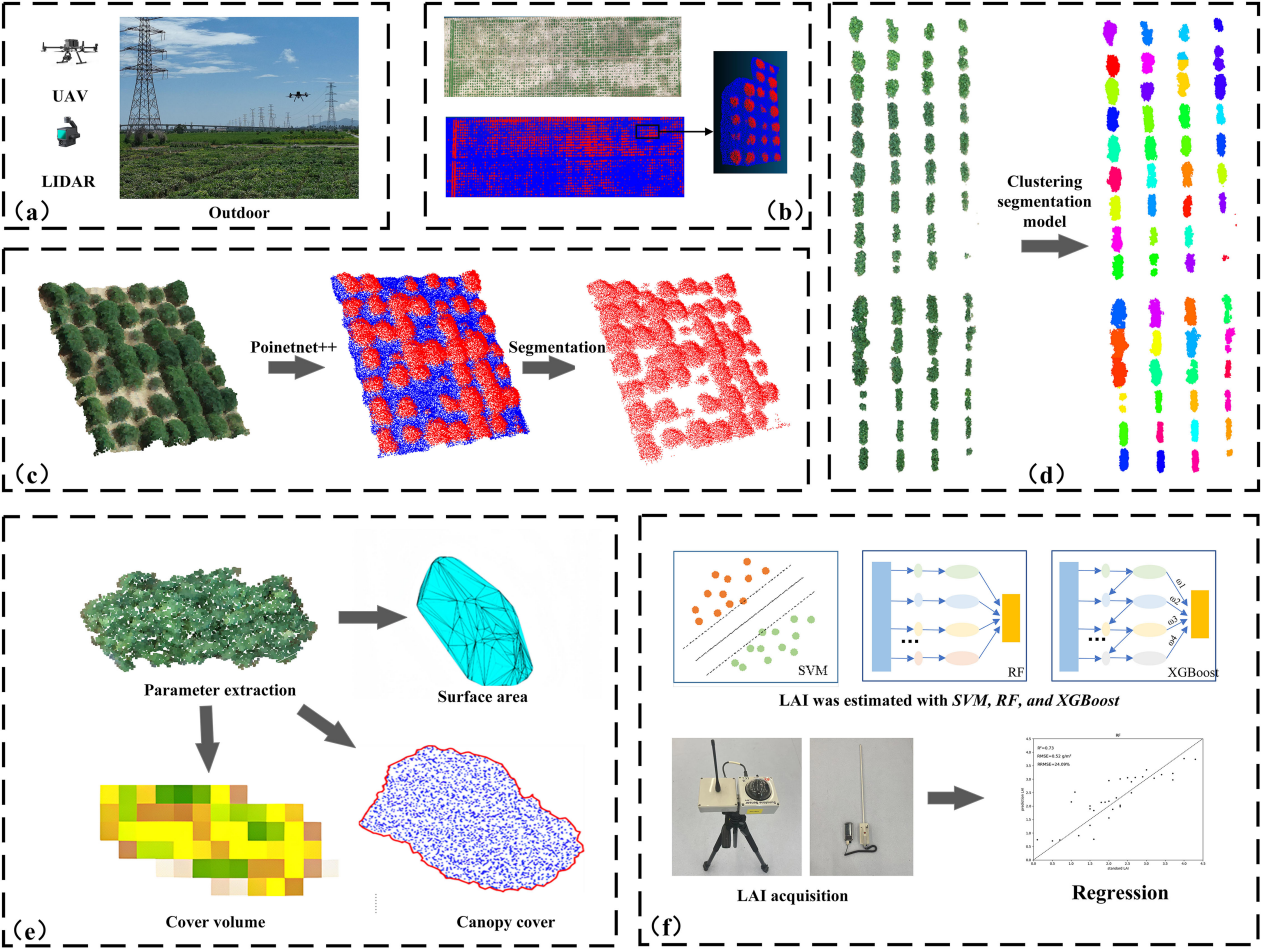
Workflow of this study: **(A)** data collection; **(B)** data preprocessing and dataset construction; **(C)** removal of natural background based on the PointNet++ segmentation model; **(D)** clustering-based segmentation of soybean plots; **(E)** plant phenotypic parameter extraction; **(F)** Prediction of Leaf Area Index Using SVM, RF, and XGBoost Models.

The methodology proposed in this study consists of five stages: material collection, removing the natural background, segmentation of individual, phenotypic parameter extraction, and prediction of leaf area index. **Figure 1** illustrates the process of LAI prediction through 3D point clouds.

### 2.2 Dataset acquisition

#### 2.2.1 Study area

**Figure 2:**
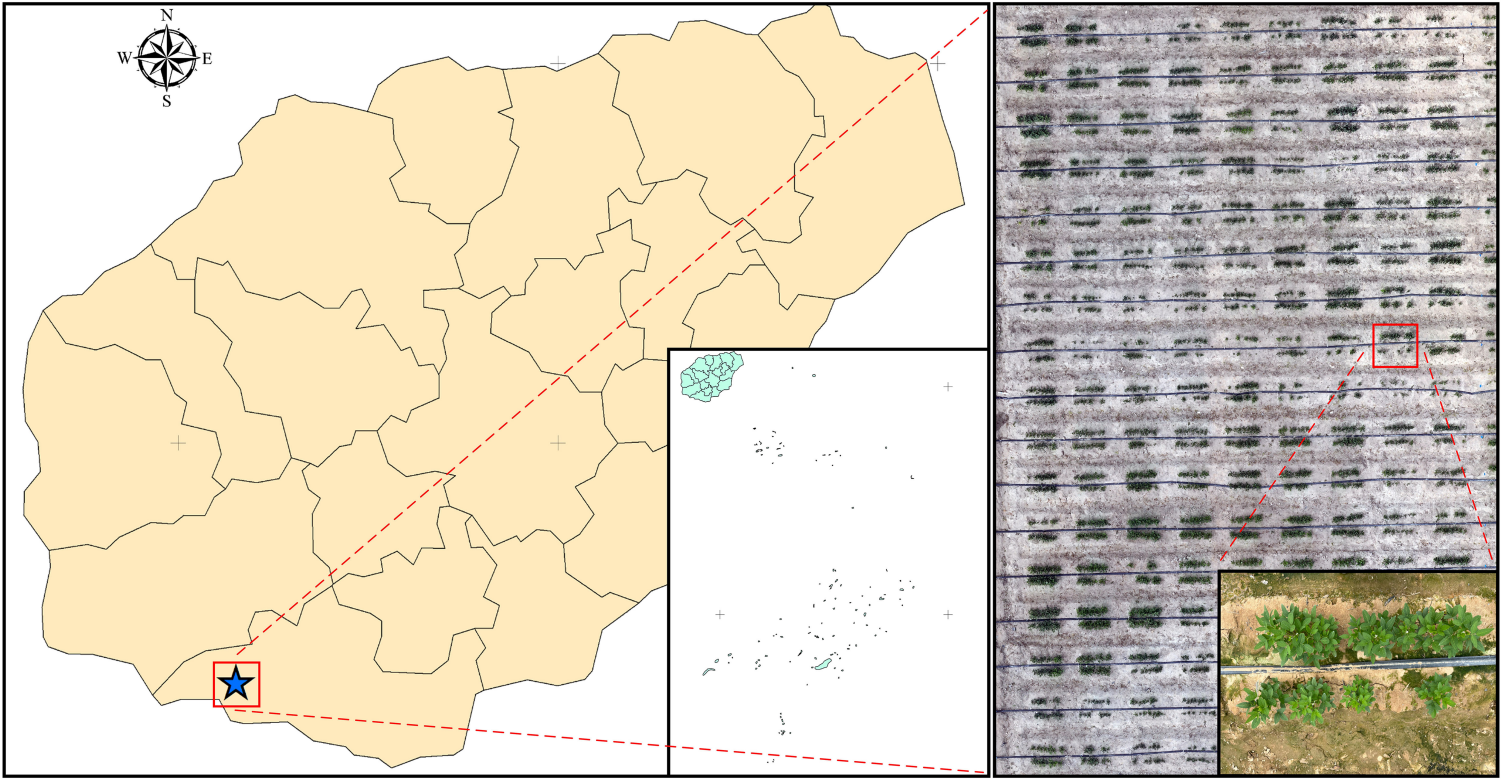
Overview of the study area.

Data were collected at the experimental site in Yazhou (**Figure 2**), Sanya, Hainan Province, China (18°21’27.11”N, 109°10’18.70”E). Soybean plants were used as the experimental material, planted using a double-row method in a plot measuring 13.0 meters in length and 4.8 meters in width, with plant spacing of 0.15 meters and ridge spacing of 0.8 meters.

In June 2022, soybean plants were sown according to this planting scheme. Point cloud data of the soybean plants were collected at 35 and 46 days after planting, corresponding to the maturity stage.

#### 2.2.2 UAV-LiDAR data

**Figure 3:**
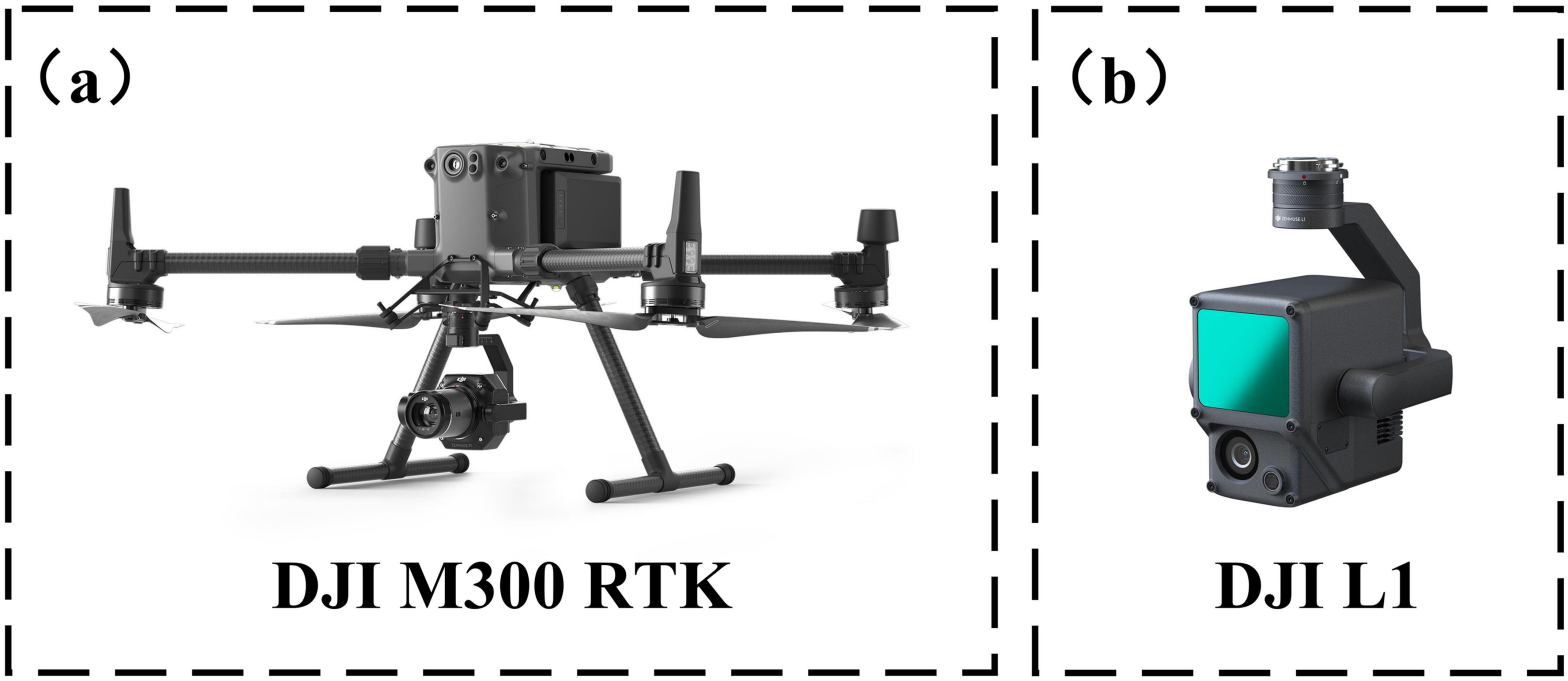
UAV platform and sensor diagram: **(A)** UAV platform; **(B)** LIDAR sensor.

LiDAR data were collected using a DJI M300 RTK UAS equipped with a Zenmuse L1 laser scanner (**Figure 3**). The Zenmuse L1 has a ranging accuracy of 3 cm, supports a maximum of three echoes, and operates with a scanning mode of repetitive scanning with a field of view (FOV) of 70.4° x 4.5°. The difference between the ground position from remote sensing equipment and the actual position, assessed by the IMU, is within 5 cm horizontally and 10 cm vertically. The scanner speed is coordinated with the UAV’s forward velocity to maintain consistent point spacing. On a scheduled date, UAV-LiDAR data were collected by flying at an altitude of 20 meters above the ground with a set speed of 6 m/s. Actual flight altitudes and speeds may have deviated slightly from these predefined values, which were calculated from subsequent flight logs. To assess the impact of flight altitude and speed on the accuracy of the acquired phenological parameters, additional flights were conducted on subsequent days at altitudes ranging from 10 to 50 meters above ground level and at programmed speeds ranging from 3 to 8 m/s. The optimal results were obtained at 20 m altitude and 6 m/s speed. Higher altitudes or faster speeds reduced point cloud density, leading to lower data accuracy, while slower speeds increased time costs. At lower altitudes, wind disturbances from the UAV’s rotors affected data quality.

#### 2.2.3 Field data collection

**Figure 4:**
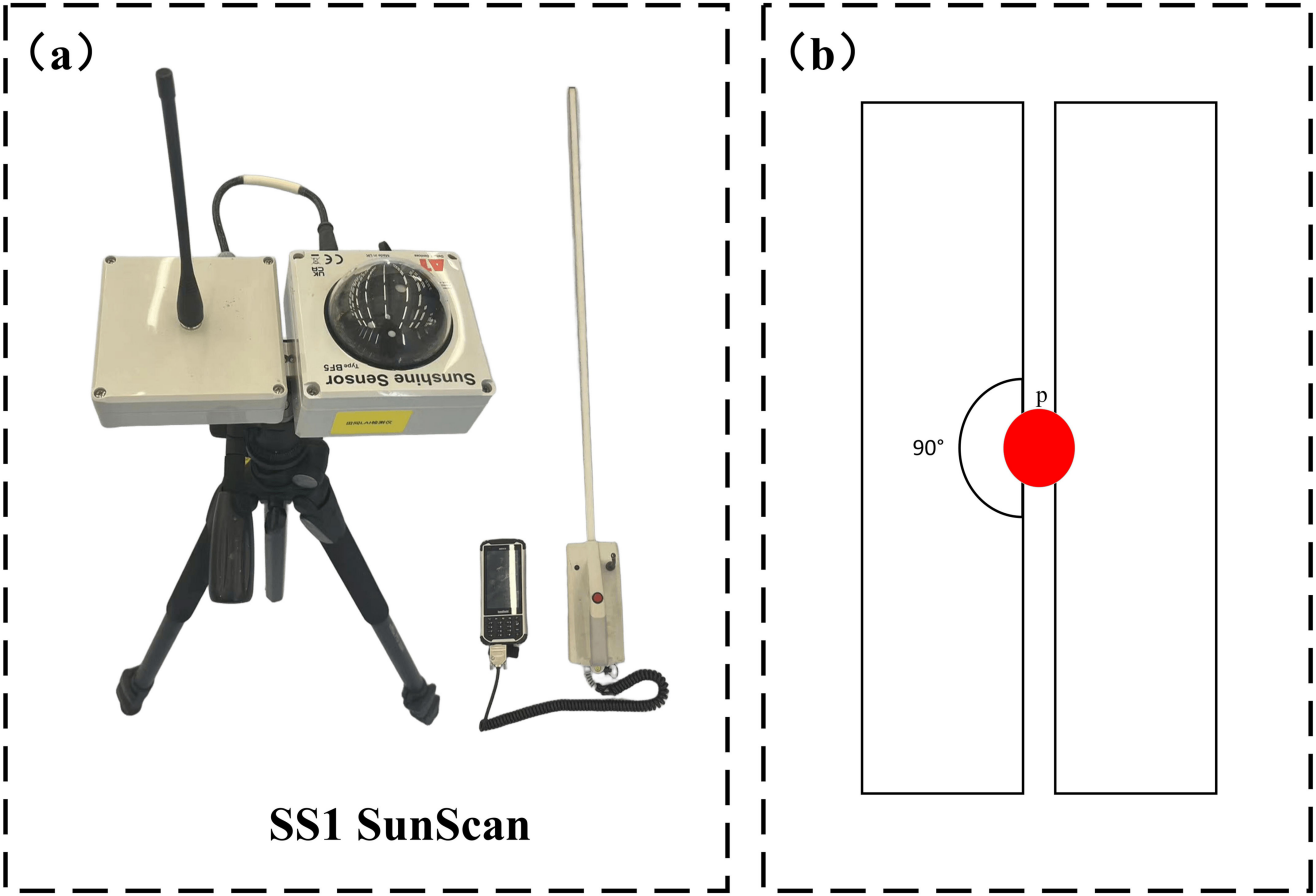
**(A)** SS1 SunScan canopy analyser used to measure leaf area index of soybean plants; **(B)** SS1 SunScan measurement orientation map.

After collecting the point cloud data, the LAI of soybeans was measured and recorded using the SS1 SunScan canopy analyzer from Dalte-T (**Figure 4**). The LAI of soybeans was determined by averaging these measurements. This portable leaf area meter features a maximum measurement width of 1 meter, with an accuracy of ±10% and a spectral response range of 400-700 nm (PAR).

To validate the estimates obtained by LiDAR, a 12 m × 4 m sampling area was established within a randomly selected three-monopoly area in the experimental field. The sampling areas were evenly distributed, and measurements were taken from multiple angles to obtain more accurate canopy height and leaf area index (LAI).

### 2.3 Data processing

#### 2.3.1 Point cloud pre-processing

The soybean point cloud generated by DJI Terra exhibits high density, with each 3D model of soybean plants in the field containing approximately 10,000 to 20,000 points. A large number of outliers within the plant model must first be manually removed, followed by the application of the StatisticalOutlierRemoval algorithm to eliminate noise. Since most outliers are challenging to remove manually, point cloud noise can be effectively filtered using appropriate filtering techniques. In terms of shape and edge preservation, the StatisticalOutlierRemoval method demonstrates superior performance, exhibiting high processing speeds and efficiently handling extensive noise. In contrast, the Statistical Filter focuses on overall statistical properties and provides superior noise reduction for smaller noise volumes.

Due to surface ambiguities of the scanned objects and external environmental noise, point cloud data may contain small fragments and discrete points different from the main point cloud. This is not conducive to point cloud extraction and matching. The StatisticalOutlierRemoval filtering method reduces noise in the original point cloud data. The principle involves calculating the mean and standard deviation for each point and its nearest N neighbors, assuming a normal distribution. Points within a pre-set range of the standard deviation (e.g., one standard deviation) are retained; otherwise, they are removed. This method effectively removes anomalously noisy points, especially when laser scanning produces an inhomogeneous point cloud. After outlier removal, the point cloud becomes smoother, facilitating the subsequent point cloud clustering process and easing convergence.

Point cloud annotation typically requires extensive manual effort (Pope and Treitz, 2013). In this study, CloudCompare software was used to process the point cloud data, which was classified into ground and vegetation regions. A dataset consisting of 126 fully annotated field soybean point cloud files was constructed.

#### 2.3.2 Removing the natural background

##### 2.3.2.1 PointNet++ segmentation model

PointNet++ (Qi et al., 2017a; Qi et al., 2017b) is a deep learning model that uses hierarchical learning to capture local features at different scales. As an enhanced version of PointNet (Qi et al., 2017a), the model focuses on fine-grained local details. In this study, the segmentation network of PointNet++ is employed for background removal from field soybean data. PointNet++ extracts local features layer by layer through local receptive fields, rather than relying solely on global point cloud information. It selects points within a specific neighborhood around the soybean plant and extracts features from these points, helping to identify the true structure of the plant while minimizing the impact of noise from weeds or soil. By focusing on the plant’s local region, PointNet++ can effectively filter out noise and capture the plant’s genuine form. Additionally, ensemble learning, by training multiple PointNet++ models on different datasets or with varied parameters, can enhance robustness by averaging predictions and reducing errors caused by noise or outliers.

**Figure 5:**
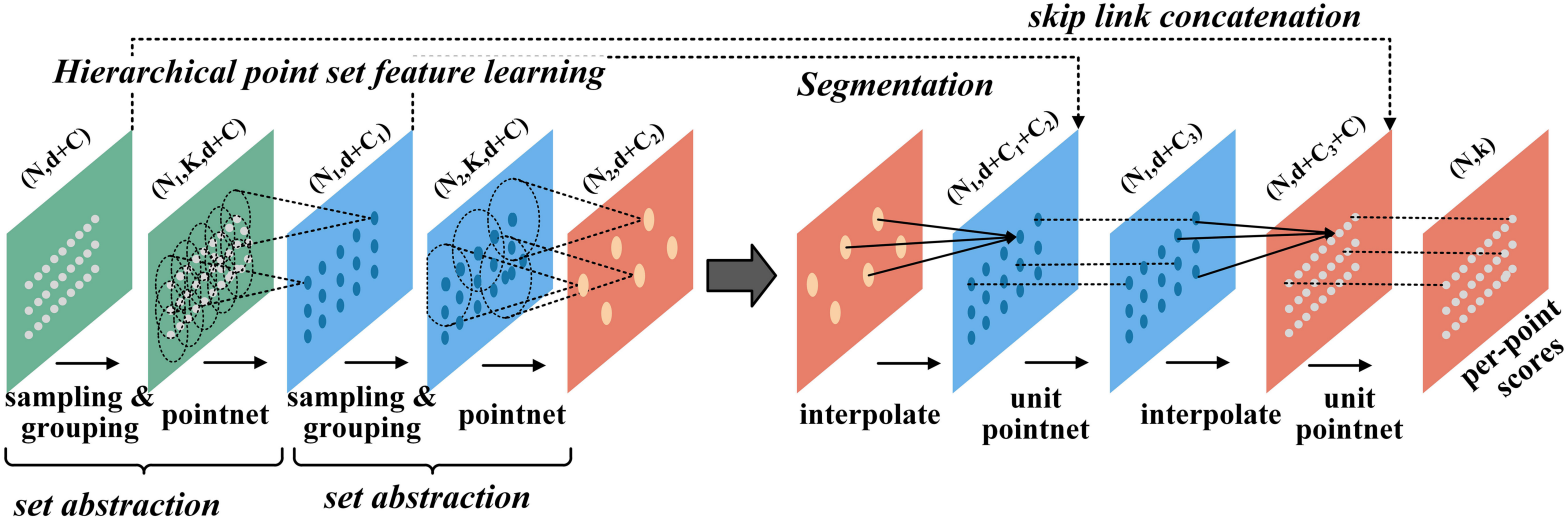
Structure of PointNet++ segmented network. n denotes the number of points, k denotes the number of groups, d denotes the coordinate dimension, and C denotes the feature dimension.

PointNet++ introduces a hierarchical structure to extract local features and uses multi-scale abstraction to progressively capture the finer details of point cloud data. Through a series of Set Abstraction (SA) layers, the network samples and groups the points, then employs multi-layer perceptrons (MLPs) and max pooling to extract local features. These local features are integrated across layers to form a global representation used for point cloud classification or segmentation. Additionally, PointNet++ enhances detail capture through upsampling, enabling efficient handling of point clouds with varying scales and densities. Its ability to learn local features makes it highly effective for point cloud processing tasks. **Figure 5** illustrates the structure of the segmentation network.

##### 2.3.2.2 Model training

The PointNet++ model was implemented using the PyTorch framework, with an initial learning rate set to 0.001. The ADAM and SGD optimizers dynamically adjusted the learning rate based on the current state of the model. The experiment was trained for 251 epochs on a Windows 11 22H2 operating system, utilizing an Intel 8th Gen processor, 256 GB of RAM, and an NVIDIA GTX 4070 GPU.

##### 2.3.2.3 Removing natural backgrounds

Segmenting plants from the background is essential for accurately assessing plant characteristics. Before segmenting individual soybean plots, they must first be separated from the background. The nature of the captured data makes it difficult to remove the soil background based on depth or color information. Paulus et al (Paulus, 2019). captured the geometric morphology of plant organs to achieve more precise segmentation. However, the high dimensionality may hinder the effective integration of spatial and color information, limiting its performance in certain applications. Rusu et al (Rusu et al., 2009). transformed point cloud features into histograms, reducing the complexity of point cloud data, thereby improving processing speed and aiding in the recognition and classification of different objects. This method utilizes differential geometric properties to generate surface histograms, ensuring density and positional invariance of surface features and suitability.

In this study, a segmentation network based on an PointNet++ model was introduced for field soybean background removal. This network facilitates the automatic point-by-point classification of soybean and soil backgrounds, improving the efficiency and accuracy of the segmentation process.

#### 2.3.3 Individual segmentation of kmeans-based clustering

Single plant segmentation using K-means clustering commences by initializing the cluster centers through the Max-Min distance algorithm, followed by K-means clustering to determine the final cluster labels. Initially, a random point in the point cloud is selected as the first clustering center, K1. The pattern sample with the maximum Euclidean distance from K1 is then chosen as the second clustering center, K2. Subsequently, for each point, the Euclidean distances to all identified cluster centers are calculated, and the smallest distance is selected for each point. If the number of pattern samples is N, N minimum distances are chosen, from which the maximum value is selected. If the total number of cluster centers has not yet been determined, the pattern sample corresponding to the maximum distance is designated as the next cluster center, Z3, and this process is iterated to identify subsequent cluster centers.

Once all cluster centers are established, the calculation step concludes. The distance from each data point to the K initialized cluster centers is computed, and the data points are assigned to the closest cluster. Once all data points are allocated, K clusters are formed. The mean of the data points within each cluster is recalculated, and this new mean becomes the updated cluster center. The distances are then recalculated for each data point relative to the updated cluster centers, and the reallocation process is repeated iteratively. The cluster centers are updated after each iteration, and the process continues until no further data points can be reassigned. Based on these initial cluster centers, subsequent K-means clustering is executed to achieve precise individual plant segmentation.

#### 2.3.4 Watershed-based monoculture segmentation

**Figure 6:**
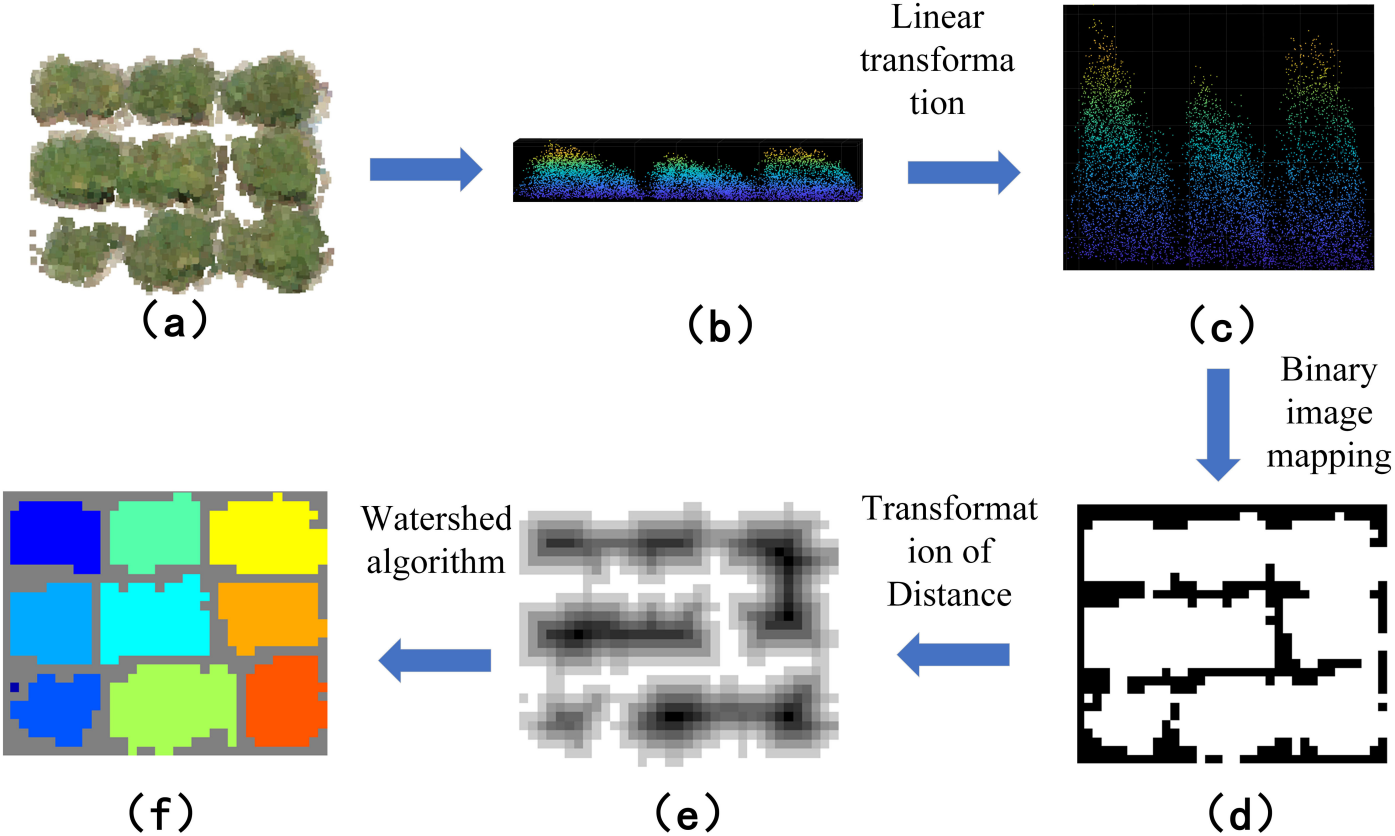
Workflow of individual segmentation based on watershed algorithm: **(A)** adhering soybean point cloud data; **(B)** point cloud side view; **(C)** linear transformation of the point cloud; **(D)** mapping of the binary image; **(E)** inversion of the binary image; **(F)** watershed segmentation of the binary map.

Watershed-based individual segmentation maps the x and y coordinates of a point cloud to a discrete 2D grid. A binary image is created by mapping the point cloud in the x and y directions. The distance to the nearest zero-valued pixel from each marked position on the grid is calculated, resulting in a greyscale image. Watershed segmentation is then performed using the negative distance transform to form the segmented regions. The point cloud data is rasterized to generate the depth image, and greyscale assignment is performed. After obtaining the greyscale image, the segmentation results are indexed back into the original point cloud data to obtain the final segmentation results of the point cloud data (**Figure 6**). The Watershed-based monoculture segmentation demonstrates superior performance in cases of severe plant overlap or irregular plant shapes, as it is better able to distinguish boundaries based on the local intensity gradients of the data.

#### 2.3.5 Estimation of soybean plant phenotypic traits

In this study, the leaf area index was estimated using an individual soybean plant point cloud model. Voxels were created by using the Octree data structure in PCL (Point Cloud Library). The number of voxels that split the point cloud along the X and Y directions was calculated based on the specified resolution. The product of the number of voxels along the X and Y directions is the total number of voxels in the first layer. The number of occupied voxel centre points is then calculated. In addition, the LiDAR beam could not penetrate the interior of the soybean region, and it was necessary to fit the missing points in the middle using the geometric features of the surrounding blank area. The value of 0.59 in Equation 2 is based on empirical experimental results.


(1) $$\text{p}=\text{int}\left(\frac{Z_{max}-Z_{min}}{VOS}\right)\;$$

(2) $$\text{k}=\frac{X_{max}-X_{min}-0.59}{VOS}\;，\text{k}=max\left(k,0\right)$$

(3) $$\text{VOG}=\sum_{p=1}^{p}\frac{2n_{1}\left(p\right)+\pi k^{2}}{2n_{T}\left(p\right)}$$

In Equation 3, VOG represents the voxel occupancy ratio, VOS represents voxel size, n_1 (p)represents the total number of voxels in the first layer, n_T (p)represents the number of body mass centers occupied by each layer. p is the number of layers of voxels along the z-axis, which is determined by the length of the whole voxel edge and the set voxel block edge.

Different voxel sizes result in different voxel forms, and in the experimental process, the voxel size should neither be too small nor too large. The minimum distance between neighboring points in the point cloud is 0.008 cm, and the maximum distance is 0.14 cm. To find the optimal voxel size, the voxel size range is set from 0.008 cm to 0.14 cm. Specifically, the voxel size is set from 0.002 cm to 0.14 cm, with an increment of 0.02 cm. Different voxel sizes were used to segment the individual point cloud, and the Pearson’s correlation coefficients between the Volume of Interest (VOI) measured using voxel blocks of different sizes and the actual measurements were calculated.

Canopy Roughness (Herrero-Huerta et al., 2020), introduced as a novel phenotypic trait, quantifies the irregularity and complexity of the canopy surface. It was calculated from high-resolution 3D point cloud data acquired by a LiDAR-equipped unmanned aircraft system (UAS). The calculation involved two key steps: first, point ambiguity was determined by analyzing the spatial relationship between each point and its neighboring points within a defined radius, using the Euclidean distance between each point and the best-fit plane of its neighborhood. This provided a local measure of surface complexity. Second, Canopy Roughness (CR) was derived by combining the interquartile range (IQR) and the median of these point ambiguities across the entire canopy. The resulting CR value, expressed in meters, effectively captures the variability and roughness of the canopy surface, offering a robust descriptor that correlates with LAI and other phenotypic traits.


(4) $$\text{CR}={\text{IQR}}^{\text{med}}$$

Plant height was determined by analyzing the Z-coordinates of the point cloud, focusing on the top 20% of the plant height to minimize ground-level disturbances. Canopy cover was estimated by projecting the 3D point cloud onto the XY plane, with the area calculated using the trapezoidal method. Canopy volume was computed by dividing the 2D projection into grid cells and summing the volume of each cell based on height differences within the grid. The canopy surface area was estimated using surface reconstruction techniques, converting the point cloud into a triangular mesh and summing the areas of the triangles. These methods provide a comprehensive approach to accurately estimate LAI and other phenotypic traits of soybean plants.

#### 2.3.6 Manual point cloud segmentation

The population soybean point cloud was read into the CloudCompare software, and the individual soybean plant point cloud was manually segmented by the polygonal point cloud segmentation method in the software as the true value of the soybean plant segmentation.

### 2.4 Evaluation metrics

The accuracy of the automatic segmentation method was assessed by comparing its results with manual annotations. The analysis was conducted at the level of individual soybean plant plots. If two or more complete plant plots were segmented into a individual plot, the segmentation was considered incorrect. Conversely, if individual plant plots were segmented correctly, the segmentation was deemed accurate. Accuracy (A) was calculated using Equation 5. Additionally, for plants that were truly segmented at the individual level, additional analysis was conducted on the number of points. Precision (P), recall (R), and F1 score (F1) were calculated using Equations 6, 7, and 8, respectively. Combining the individual level and point cloud level analyses, the precision multiplied by the F1 score (A * F1) was used to comprehensively evaluate the soybean plot segmentation.


(5) $$\text{A}=\frac{\text{T}{\text{P}}_{\text{C}}}{\text{A}{\text{C}}_{\text{p}}}$$

where A, and are the accuracy, the number of truly segmented plants, and the actual number of plants, respectively.


(6) $$P=\frac{TP_{C}}{TP_{C}+FP_{C}}\;$$

(7) $$R=\frac{TP_{C}}{TP_{C}+FN_{C}}\;$$

(8) $$F_{1}=\frac{2PR}{P+R}\;$$

Where P, R, and F1 represent precision, recall, and F1 score, respectively. The other terms refer to the number of points correctly assigned to the corresponding soybean plants, the number of points incorrectly assigned to the corresponding soybean plants, and the number of points misassigned to other soybean plants.

In the Remove Natural Background section, the classification accuracy was validated using manually segmented data and calculated using the following formula:


(9) $$Accuracy=\frac{TP+TN}{TP+TN+FP+FN}\;$$

True Positive (TP), True Negative (TN), False Positive (FP), and False Negative (FN) are the four basic metrics used to assess the accuracy of a classification algorithm.TP refers to the number of points that the algorithm correctly identifies as plants, TN is the number of points that the algorithm correctly identifies as non-plants. FP is the number of points that the algorithm incorrectly labeled non-plants as plants, and FN is the number of points where the algorithm incorrectly labeled plants as non-plants.

## 3 Results and discussion

### 3.1 Removal of natural background

**Figure 7:**
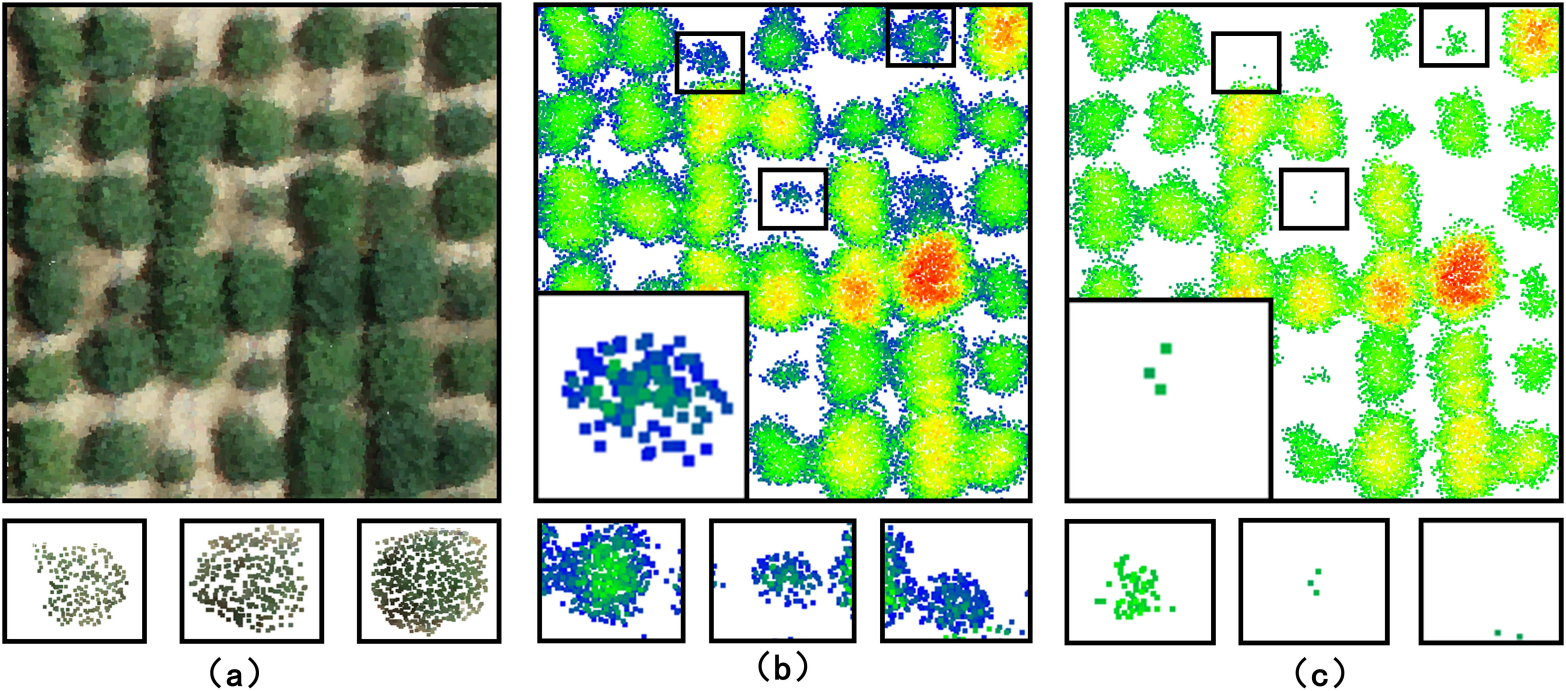
Field-grown soybean plants natural background removal Typical examples of natural background processing results are **(A)** RGB point cloud image **(B)** removal results after pointnet++ natural background processing **(C)** removal results after ransac natural background processing.

Before phenotypic analysis, the natural background of soybean plants needs to be removed. The accuracy of background removal was assessed by analyzing the extent to which soybean plants were successfully extracted from the original point cloud data after background elimination. Automatic point-by-point classification of soybean plant and background was performed using techniques based on an PointNet++ model. **Figure 7** illustrates the results of natural background removal for field-grown soybean plants. The first column presents the RGB image of the soybean fields captured by our platform, while the second and third columns show point clouds of soybean plants extracted from the background.

Based on the point cloud data of the soybean fields, the dataset was partitioned into 6x7 soybean plots for subsequent analysis. The PointNet++ segmentation model was compared with the RANSAC algorithm-based segmentation model using 6x7 soybean plots as samples. According to Formula (9), the segmentation accuracy of the PointNet++ model exceeds that of the RANSAC algorithm by 6.73%.

### 3.2 Individual segmentation results

**Figure 8:**
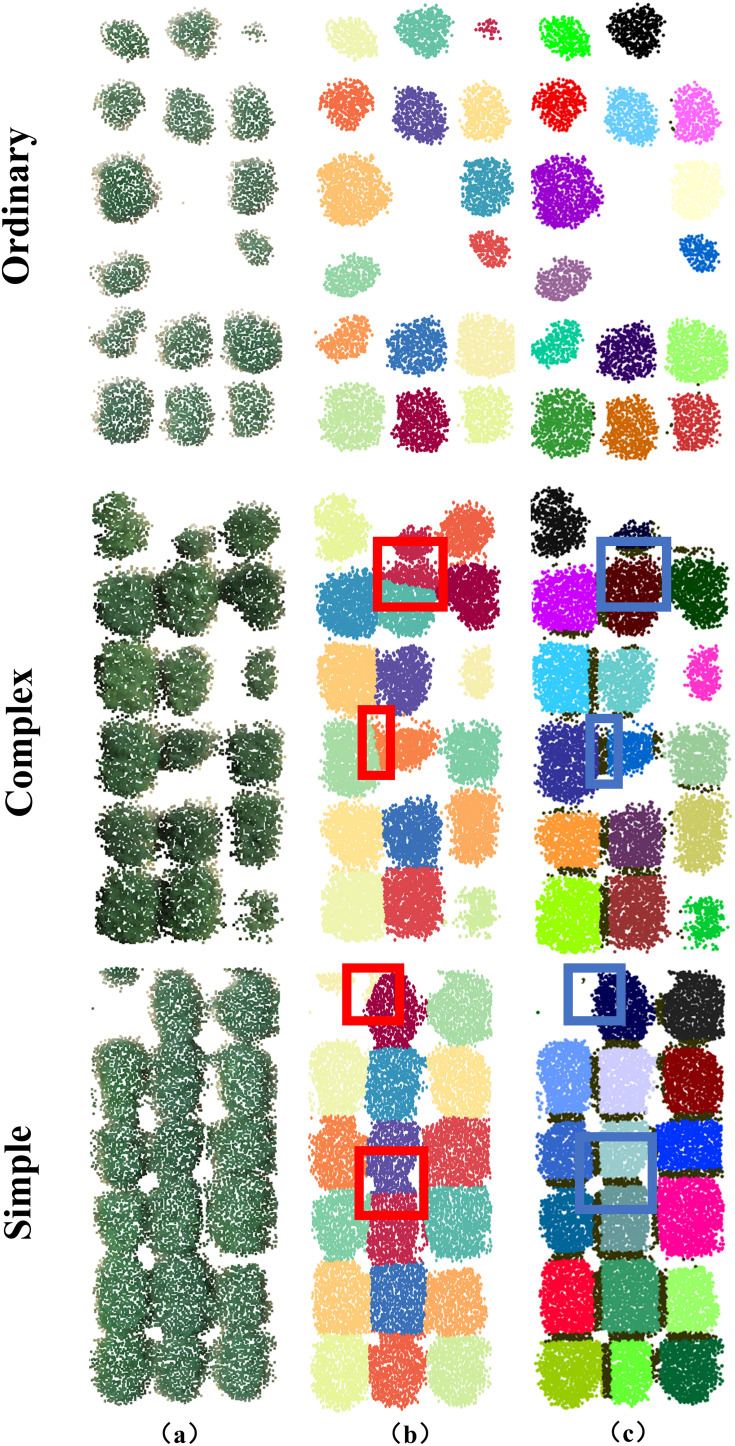
Visualisation of point cloud segmentation using kmeans-based algorithm and watershed-based algorithm. The first, second and third rows are one of the simple, normal and complex plant-type structures, respectively. **(A)** is a field soybean plant to be segmented. **(B)** is the segmentation result of the kmeans-based algorithm. Red circled regions indicate incorrect segmentation. Blue circled regions indicate correct segmentation. Contact points between each soybean plot are indicated by black dots. **(C)** Segmentation results based on the watershed algorithm.

To verify the individual segmentation results for soybean plants with different complex growth layouts, the plots were classified into three categories based on the degree of adhesion between them: simple (no overlapping regions), ordinary (one overlapping region with a low degree of adherence), and complex (completely overlapping regions with a low degree of adherence). The quantities of the three plant types for evaluation are 15, 18, and 9, respectively, with examples shown in **Figure 8**.

Segmentation of the point cloud of 42 soybean plots using kmeans-based algorithm and watershed-based algorithm, as shown in **Figure 8**. Visual inspection indicated that the watershed-based algorithm produced a more complete soybean point cloud compared to the kmeans-based algorithm (**Figures 8A–C**).

The k-means clustering algorithm was used to evaluate the performance of the watershed-based segmentation method. The results demonstrated that this method can effectively segment the plots (**Figure 8B**). However, when soybean plots adhesion complexity was high, the k-means algorithm performed poorly (**Figure 8C**). Specifically, the k-means algorithm failed to accurately segment each plot from multiple adherent soybean plots (**Figure 8B**). The watershed-based segmentation method performed significantly better, each contact point in the localized region will separate different clustering results, thus completing the segmentation. particularly in completely segmenting each complex adhesion (**Figure 8C**). The k-means algorithm struggled with recognizing edge and height features with a high degree of adhesion, resulting in substantial segmentation boundary errors (**Figure 8B**).

**Figure 9:**
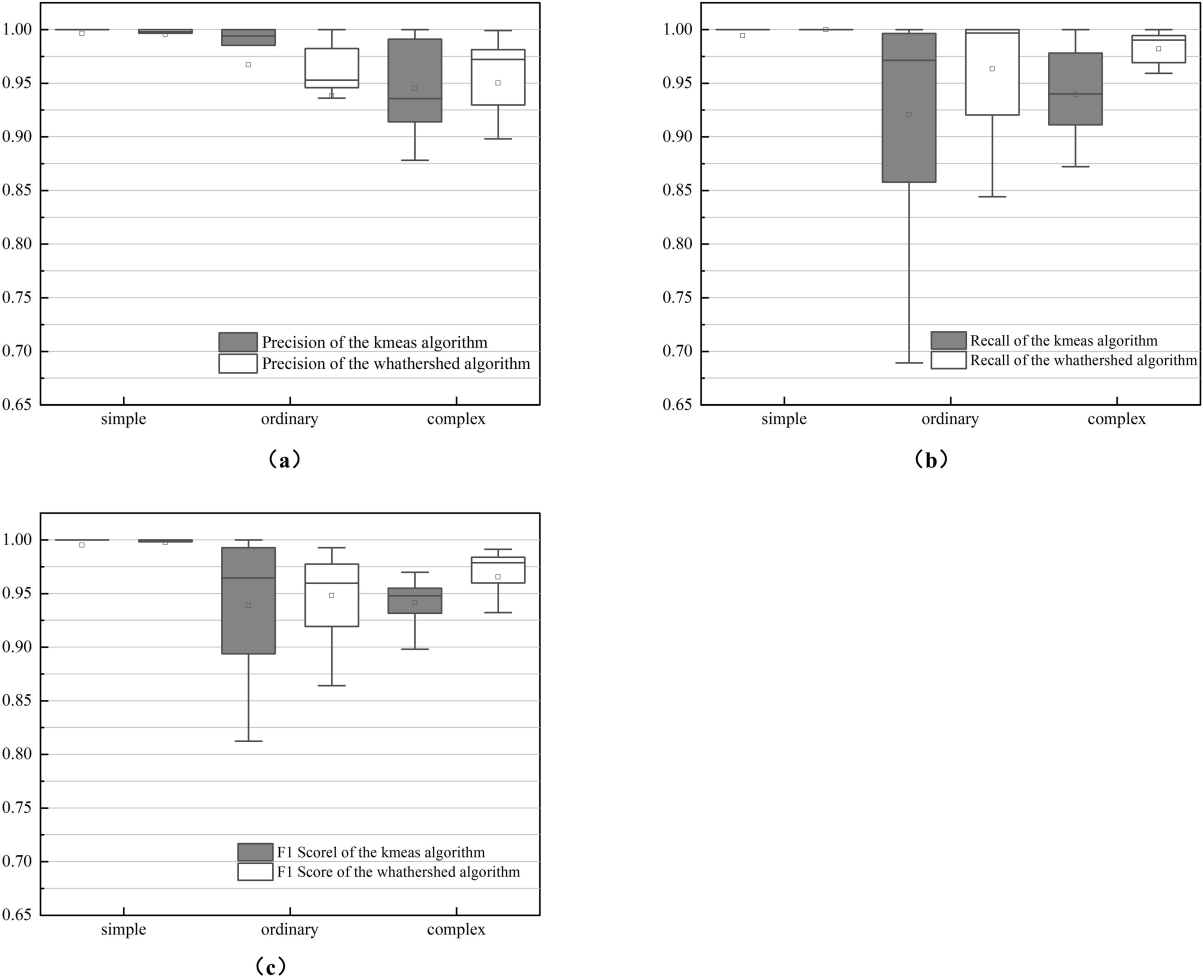
Box plot of precision metrics between k-means and watershed-based algorithms: **(A)** is recall; **(B)** is precision; **(C)** is F1 score.

The performance of the two methods was evaluated using manually segmented point clouds (**Table 1**). T-tests were performed to evaluate the mean precision, recall, and F1 scores between the two algorithms (**Table 2**). The results in **Table 1** are further illustrated with box-and-line plots (**Figure 9**). For the watershed algorithm, the average F1 scores across the three types of soybean plants varied between 0.89 and 0.90, with average precision values spanning 0.95 to 0.96, and recall values between 0.85 and 0.86. The increased mean recall value and slight decrease in standard deviation compared to the k-means clustering algorithm suggest better segmentation in plot segmentation.

**Table 1:** Mean and standard deviation of precision, recall and F1 score values using k-means versus watershed-based algorithms.

Plant category	Simple			Ordinary			Complex		
Precision indicators	p	r	F1	p	r	F1	p	r	F1
Kmeans clustering algorithm	0.9964	0.9943	0.9952	0.9671	0.9206	0.9388	0.9452	0.9398	0.9412
Watershed-based clustering algorithm	0.9955	1	0.9977	0.9384	0.9632	0.9482	0.9502	0.982	0.9654

All results are calculated based on manual segmentation. p, precision; r, recall; f1, F1-Score.

**Table 2:** t-test for precision, recall and F1 score using k-means with watershed-based algorithm.

Plant category	Simple			Ordinary			Complex		
Precision indicators	p	r	F1	p	r	F1	p	r	F1
t	0.516	-1	-0.838	2.133	-2.339	-0.79	-0.48	-4.038	-3.231
p	0.62	0.347	0.426	0.048*	0.032*	0.44	0.638	0.001*	0.006*

* indicates a significance level of 0.05.

### 3.3 Comparison of LAI estimation models

After combining UAV-derived plant traits with machine learning algorithms, the feature selection showed voxel occupancy ratio to be superior to other features. Finally, six vegetation traits (plant height, Canopy Roughness, surface area, volume, cover, and voxel occupancy ratio) were selected to construct LAI prediction model using three machine learning algorithms: RF, SVM, and XGBoost.

Data from 35 and 46 days after soybean sowing in the field were divided into training and test sets in the ratio of 3:7. The LAI estimation model was constructed using vegetation phenotypes as multivariate input variables. Among the modeling methods, the RF model performed the best in the calibration set (R² = 0.89, RMSE = 0.22, RRMSE = 0.1373), followed by the SVM model (R² = 0.73, RMSE = 0.52, RRMSE = 0.2406). In the validation set, the SVM model performed best (R² = 0.79, RMSE = 0.47, RRMSE = 0.2183), while the RF model’s R² dropped from 0.89 to 0.73, with a 20.18% increase in RMSE and a 10.33 increase in RRMSE. The XGBoost model performed slightly worse than the other two, but still maintained good precision (R² > 0.69, RMSE< 0.65). These results indicate that the SVM model offers the best estimation accuracy and stability, with the other two models also yielding strong predictive performance. SVM outperformed both RF and XGBoost in validation, particularly due to its ability to model complex, nonlinear relationships in high-dimensional data. While RF and XGBoost are robust ensemble methods, their reliance on tree structures requires additional tuning to effectively capture such complexities. Moreover, SVM’s inherent feature selection minimizes overfitting, enhancing model accuracy and explaining its superior performance in our study.

**Figure 10:**
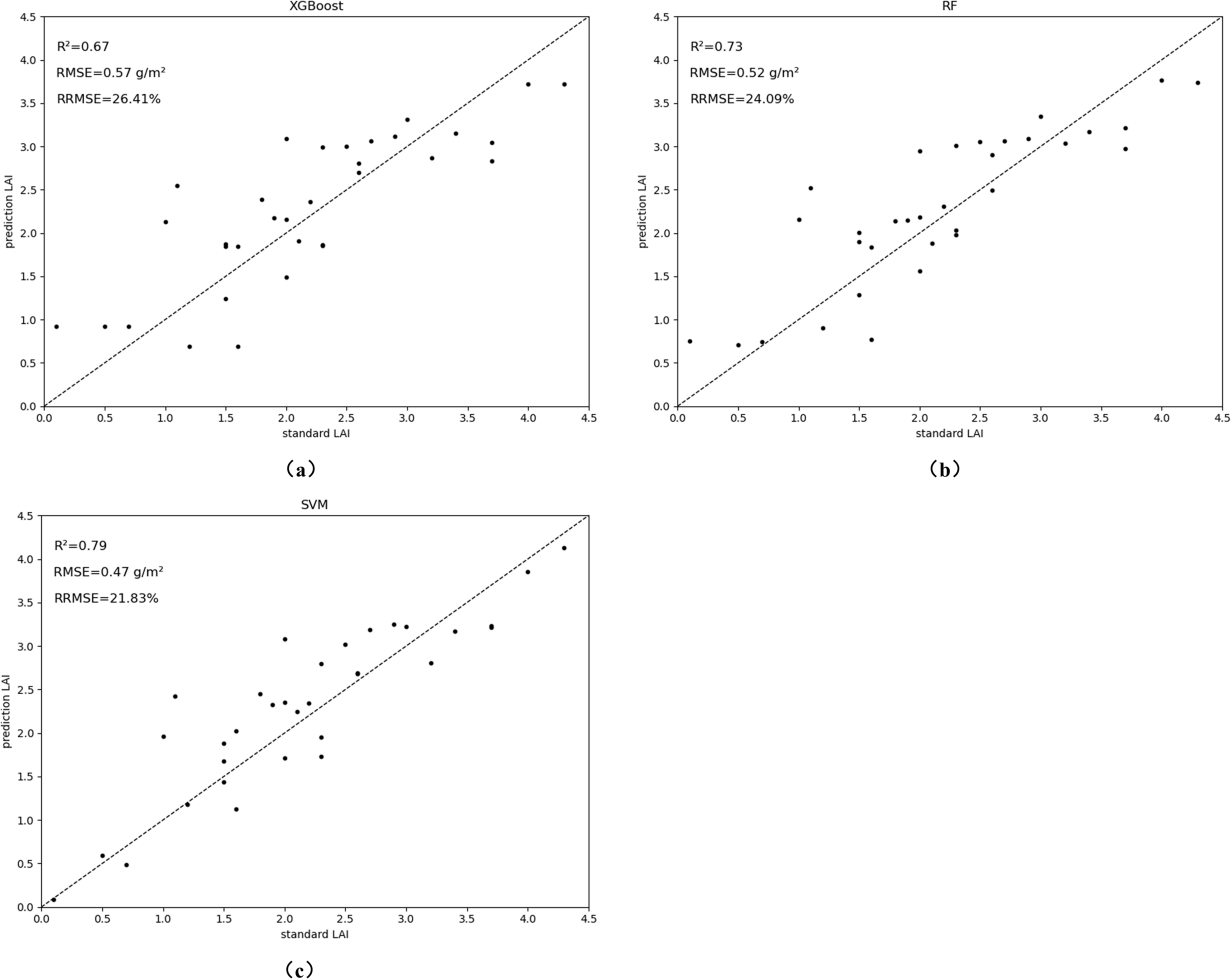
Accuracy evaluation results of LAI estimation models. The models evaluated are: **(A)** XGBoost; **(B)** RF; **(C)** SVM.

The scatter plot in **Figure 10** shows that the predicted LAI values are similar to the actual values, with RMSE between 0.47 and 0.57, and RRMSE between 0.2183 and 0.2641. Incorporating canopy phenotypic traits, optimal estimates were achieved using three different machine learning models. Although this study focuses on soybean, the methodology can be adapted to other crops with similar growth patterns, such as maize, wheat, or rice. To improve scalability, future work could integrate additional remote sensing data, like multispectral or hyperspectral imagery, for enhanced accuracy. Optimizing the computational efficiency of the pipeline would also enable large-scale agricultural monitoring across different crops and environments.

## 4 Conclusions

In summary, this study proposed an automated pipeline combining deep learning and clustering algorithms for individual segmentation and LAI estimation in field-grown soybean plants. The The PointNet++ model significantly improved background segmentation, achieving an IOU of 0.86 and an accuracy of 0.95. The clustering algorithm effectively addressed challenges in individual segmentation, particularly in adhesion regions. The estimated LAI showed a strong correlation with measurements (R²=0.88). This method offers an efficient approach for monocot segmentation and plant phenotyping, particularly beneficial for soybean breeding. The results demonstrate the method’s high accuracy and potential for automated, high-precision LAI extraction in precision agriculture. This method could significantly impact farmers by enabling efficient, large-scale monitoring of crop health and growth. Automated LAI estimation provides timely insights into crop development, optimizing resource management and supporting informed decisions to maximize yield and sustainability.

## Data Availability

The datasets presented in this article are not readily available because data available on request from the authors. Requests to access the datasets should be directed to shibingbiehuang@163.com.
